# ﻿*Sedum qingyuanense* (Crassulaceae), a new species from Qingyuan, Guangdong, China

**DOI:** 10.3897/phytokeys.268.174287

**Published:** 2025-12-15

**Authors:** Xiao-Wei Yi, Zhi-Yi Xie, Xue-Gui Shen, Wei Xu, Qiu-Gen Zeng, Ke Tang, Yan-Shuang Huang, Qiang Fan

**Affiliations:** 1 State Key Laboratory of Biocontrol and Guangdong Provincial Key Laboratory of Plant Stress Biology, School of Life Sciences, Sun Yat-sen University, Guangzhou 510275, China Sun Yat-sen University Guangzhou China; 2 Guangdong Ecological and Environmental Monitoring Center, Guangzhou 510308, China Guangdong Ecological and Environmental Monitoring Center Guangzhou China; 3 Qingyuan Forestry Bureau, Qingyuan 511518, China Qingyuan Forestry Bureau Qingyuan China

**Keywords:** New species, nrITS, phylogeny, *
Sedum
*

## Abstract

This study describes a new species of *Sedum* (Crassulaceae), *Sedum qingyuanense***sp. nov.** discovered in Qingyuan City, Guangdong Province, China. Morphologically, *S. qingyuanense* resembles *S. alfredii* and *S. emarginatum*. The new species may have previously been misidentified as *S. alfredii*. However, the new species can be readily distinguished from *S. alfredii* by its creeping sterile stems, opposite leaves, emarginate leaf apices, and smaller sepals. Compared to *S. emarginatum*, it is differentiated by its creeping sterile stems, smaller sepals, and conspicuous pseudopetioles. Phylogenetic analysis based on the nuclear ribosomal DNA internal transcribed spacer (ITS) region revealed that the new species forms a sister clade to *S. emarginatum* within Sedum sect. Sedum, supported by moderate to strong bootstrap support (> 80%). Integrating both morphological evidence and the phylogenetic tree, we establish it as an independent lineage.

## ﻿Introduction

The genus *Sedum* L. (Crassulaceae), comprising approximately 474 accepted species (WFO 2025), represents the most species-rich genus within the family. It has a broad distribution across temperate and subtropical regions of the Northern Hemisphere, with biodiversity hotspots concentrated in the Mediterranean Basin, Central America, the Himalayas, and East Asia (Stephenson 1994; Thiede and Eggli 2007). A few species extend into the Southern Hemisphere, particularly in Africa and South America. *Sedum* species typically exhibit herbaceous to subshrubby growth forms with succulent leaves and stems, an adaptation to xeric habitats such as deserts, cliffs, rock crevices, and sandy slopes (Thiede and Eggli 2007). Certain taxa, including *Sedum lineare* Thunb. and *S. sarmentosum* Bunge, are widely used in urban green roofing due to their compact growth habit, shallow root systems, stress tolerance, low maintenance requirements, and ornamental value (Schindler et al. 2019).

East Asia constitutes one of the major centers of *Sedum* diversity (Thiede and Eggli 2007; Ito et al. 2017a). According to the Flora of China (Fu and Ohba 2001), 121 *Sedum* species occur in China, of which 91 are endemic. These species are classified into three sections: sect. Oreades Fröd., sect. Sedum, and sect. Filipes Fröd., with the majority primarily distributed in southwestern China. Among them, Sect. Sedum represents the most species-rich group (Zou et al. 2025). It is characterized by convex ventral surfaces of carpels and follicles.

From 2005 to 2024, 18 new species of the genus *Sedum* have been described from China (Dai et al. 2025). Between 2024 and October 2025, an additional five species—*S. orientalichinense* Q. Fan & P. Li (Dai et al. 2025), *S. guangxiense* Yan Liu & C.Y.Zou (Zou et al. 2025), *S. simingshanense* Y.L.Xu (She et al. 2025a), *S. yongkangense* Y.L.Xu et Z.H.Chen (She et al. 2025b), and *S. shunhuangense* L.Wu & Z.L.Feng (Feng et al. 2025) —were published. As of 2025, the genus *Sedum* comprises 144 species in China, including 114 endemic species. During field surveys in Guangdong Province, southern China, this species was discovered growing on rocky slopes in Qingyuan City. The species morphologically resembles *S. alfredii* Hance and *S. emarginatum* Migo but is distinguished by its smaller sepals, creeping sterile stems, and conspicuous pseudopetioles. Phylogenetic analysis based on nrITS sequencing supports its status as an independent lineage. Morphological comparisons and molecular evidence confirm that *S. qingyuanense* represents a previously undescribed species within Sedum sect. Sedum. This paper provides a comprehensive morphological description, taxonomic discussion, and ecological illustrations of the new species.

## ﻿Methods

### ﻿Morphological study

To obtain fresh material for morphological analysis, detailed field investigations were conducted during the flowering period of the new species and its close relatives: *S. emarginatum* (Hangzhou), *S. alfredii* (Hangzhou and Guangzhou), *S. jinglanii* (ShaoGuan). Collected 4 specimens were thoroughly examined, dissected, and photographed. Living specimens of this new species were collected from TaiHe Grotto, Qingyuan City, Guangdong Province, China.

Digital specimens of related species were examined via the Chinese Virtual Herbarium (https://www.cvh.ac.cn/); and the Global Biodiversity Information Facility (https://www.gbif.org/) to obtain comparative morphological data. The holotype was collected from TaiHe Grotto, Qingyuan City, and is deposited in the Herbarium of Sun Yat-sen University (**SYS!**).

### ﻿Molecular study

Fresh leaves from two individuals of *S. qingyuanense* were collected at TaiHe Grotto, Qingyuan City, and dried in silica gel. Total DNA was extracted using a modified CTAB method (Doyle and Doyle 1987). The partial internal transcribed spacer 1 (ITS1), the 5.8S ribosomal RNA gene, and the partial internal transcribed spacer 2 (ITS2) region were amplified using the primers ITS1 and ITS4 (White et al. 1990), following PCR protocols from Huang et al. (2021).

We downloaded 74 ITS sequences from GenBank, representing 53 *Sedum* species (including subspecies and variants) and 3 outgroups (Table 1). *Greenovia aizoon*, *Aeonium viscatum* and *A. lancerottense* were selected as outgroups (Huang et al. 2023). Ultimately, a total of 79 sequences were used to construct the phylogenetic tree, including the sequence of *S. qingyuanense* and our newly sequenced *S. alfredii* and *S. emarginatum* sequences.

**Table 1.** Taxa, voucher information, GenBank accession numbers and references for ITS sequences of Sedum (S.) species and three outgroups used for phylogenetic analyses in this study.

Taxon	Voucher	Accession number	Reference
*S. actinocarpum*	Ito 1749	LC229265	Ito et al. 2017a
*S. alfredii*	IBK194564	FJ919949	Wang and Shu unpublished
	IBK114924	FJ919951	Wang and Shu unpublished
	–	PX560094	**Present Study**
*S. arisanense*	Ito 1842	LC229273	Ito et al. 2017a
	Ito 1836	LC229272	Ito et al. 2017a
*S. baileyi*	LBG0064555	FJ919935	Wang and Shu unpublished
*S. bergeri*	–	AY352897	Ni et al. unpublished
*S. boninense*	Ito 2371	LC229242	Ito et al. 2017a
*S. bulbiferum*	Ito 416	LC229234	Ito et al. 2017a
	130514hs41	KM111166	Xie et al. 2014
	130524qz09	KM111165	Xie et al. 2014
S. brachyrinchum var. brachyrinchum	Ito 1359	LC229274	Ito et al. 2017a
*S. danjoense*	Ito 3658	LC260127	Ito et al. 2017b
*S. emarginatum*	130512hs27	KM111145	Xie et al. 2014
	130529hz03	KM111146	Xie et al. 2014
	130503jz21	KM111147	Xie et al. 2014
	24041001	PP981038	Dai et al. 2025
	24052201	PP981037	Dai et al. 2025
	AYJT-Nanjing-0308-08	EU592006	–
	–	PX560095	**Present Study**
	–	PX560096	**Present Study**
*S. erici-magnusii*	Ito 2077	LC229235	Ito et al. 2017a
*S. erythrospermum*	Tsutsumi 1504	AB906473	Ito et al. 2014b
*S. formosanum*	Ito1115	LC530813	Ito et al. 2020
	Ito2296	LC260124	Ito et al. 2017b
*S. hakonense*	Mayuzumi C00005	AB088625	Mayuzumi and Ohba 2004
*S. hangzhouense*	Ito 2604	LC260130	Ito et al. 2017b
*S. japonicum*	Kokubugata 16749	AB906475	Ito et al. 2014b
S. japonicum var. oryzifolium	Ito 2285	LC229239	Ito et al. 2017a
S. japonicum var. pumilum	Ito 2287	LC229240	Ito et al. 2017a
S. japonicum var. senanense	Ito 2200	LC229238	Ito et al. 2017a
*S. jinglanii*	Y. S. Huang 21040301	OP288035	Huang et al. 2023
	DNPC 2873	OQ162326	Huang et al. 2023
*S. jiulungshanense*	Ito 76	LC229243	Ito et al. 2017a
*S. kiangnanense*	CMQ1030	LC229244	Ito et al. 2017a
*S. lineare*	Mayuzumi C00120	AB088623	Mayuzumi and Ohba 2004
*S. lipingense*	ZRB1479	MN150061	Zhang et al. 2019
*S. lungtsuanense*	Ito 3563	LC260131	Ito et al. 2017b
*S. makinoi*	Kokubugata 16730	AB906476	Ito et al. 2014b
*S. mexicanum*	Ito 647	LC229247	Ito et al. 2017a
*S. microsepalum*	Ito 2771	LC229282	Ito et al. 2017a
	Ito 1965	LC229281	Ito et al. 2017a
*S. morrisonense*	Ito 2765	LC229290	Ito et al. 2017a
	Hornat S1	LM993281	Nikulin et al. 2016
*S. multicaule*	Miyamoto et al. TI9596136	AB088631	Mayuzumi and Ohba 2004
*S. nagasakianum*	Ito 2064	LC229249	Ito et al. 2017a
*S. nokoense*	Kokubugata 10426	AB906478	Ito et al. 2014b
*S. oligospermum*	Ito 74	LC229250	Ito et al. 2017a
*S. oreades*	Rao 090803-03	KF113733	Zhang et al. 2014
S. polytrichoides subsp. polytrichoides	CMQ1057	LC229251	Ito et al. 2017a
S. polytrichoides var. setouchiense	Ito 2298	LC229253	Ito et al. 2017a
*S. qingyuanense*	TH1001	PX560092	**Present Study**
	TH1002	PX560093	**Present Study**
*S. rupifragum*	Ito2070	LC229254	Ito et al. 2017a
*S. sarmentosum*	Ito 978	LC229255	Ito et al. 2017a
*S. satumense*	Ito 2295	LC229256	Ito et al. 2017a
*S. sekiteiense*	Ito 1456	LC229295	Ito et al. 2017a
*S. simingshanense*	-	PP464049	She et al. 2025
	-	PP464048	She et al. 2025
*S. subtile*	Ito 624	AB930277	Ito et al. 2014a
	Shimizu 1999	AB088622	Mayuzumi and Ohba 2004
*S. taiwanianum*	Ito 2770	LC229297	Ito et al. 2017a
	Ito 2770	LC229296	Ito et al. 2017a
*S. tetractinum*	Ito 3623	LC260135	Ito et al. 2017b
*S. tianmushanense*	Ito 355	LC229261	Ito et al. 2017a
*S. tosaense*	Kokubugata 16726	AB906483	Ito et al. 2014b
*S. triactina*	9596091	AB088629	Mayuzumi and Ohba 2004
*S. tricarpum*	Ito 2269	LC229259	Ito et al. 2017a
	-	PP989617	-
*S. trullipetalum*	Miyamoto et al. 9420132	AB088630	Mayuzumi and Ohba 2004
*S. truncatistigmum*	Ito 3254	LC229306	Ito et al. 2017a
*S. xunvense*	-	PV101948	Chai et al. 2024
	-	PV101947	Chai et al. 2024
*S. yabeanum*	Ito 396	AB906490	Ito et al. 2014b
*S. zentaro-tashiroi*	Ohba 1998	AB088619	Mayuzumi and Ohba 2004
**Outgroups**			
*Aeonium lancerottense*	Mort 1518	AY082143	Mort et al. 2002
*Aeonium viscatum*	Mort 1432	AY082154	Mort et al. 2002
*Greenovia aizoon*	Mort 1425	AY082112	Mort et al. 2002

Sequence alignment was performed using MAFFT v7.505 (Katoh and Standley 2013) with the ‘--auto’ strategy. The initial alignment was manually inspected and refined in BioEdit v7.2.5 to correct misalignments in hypervariable regions. Maximum Likelihood (ML) phylogenetic inference was conducted using IQ-TREE 2.1.4 (Minh et al. 2020). The best-fit nucleotide substitution model was determined through the built-in ModelFinder utility, which selected the SYM+I+G4 model under the Bayesian Information Criterion (BIC). Branch support was assessed through standard non-parametric bootstrap analysis with 3,000 replicates. Bayesian inference (BI) analysis was conducted with MrBayes v.3.2.1 (nruns = 2, nchains = 4, mcmc = 30,000,000). The consensus tree was visualized and annotated in FigTree v1.4.4, with branches scaled by the expected number of substitutions per site and bootstrap support values (BS) displayed at the nodes.

## ﻿Results and discussion

**Figure 1.**
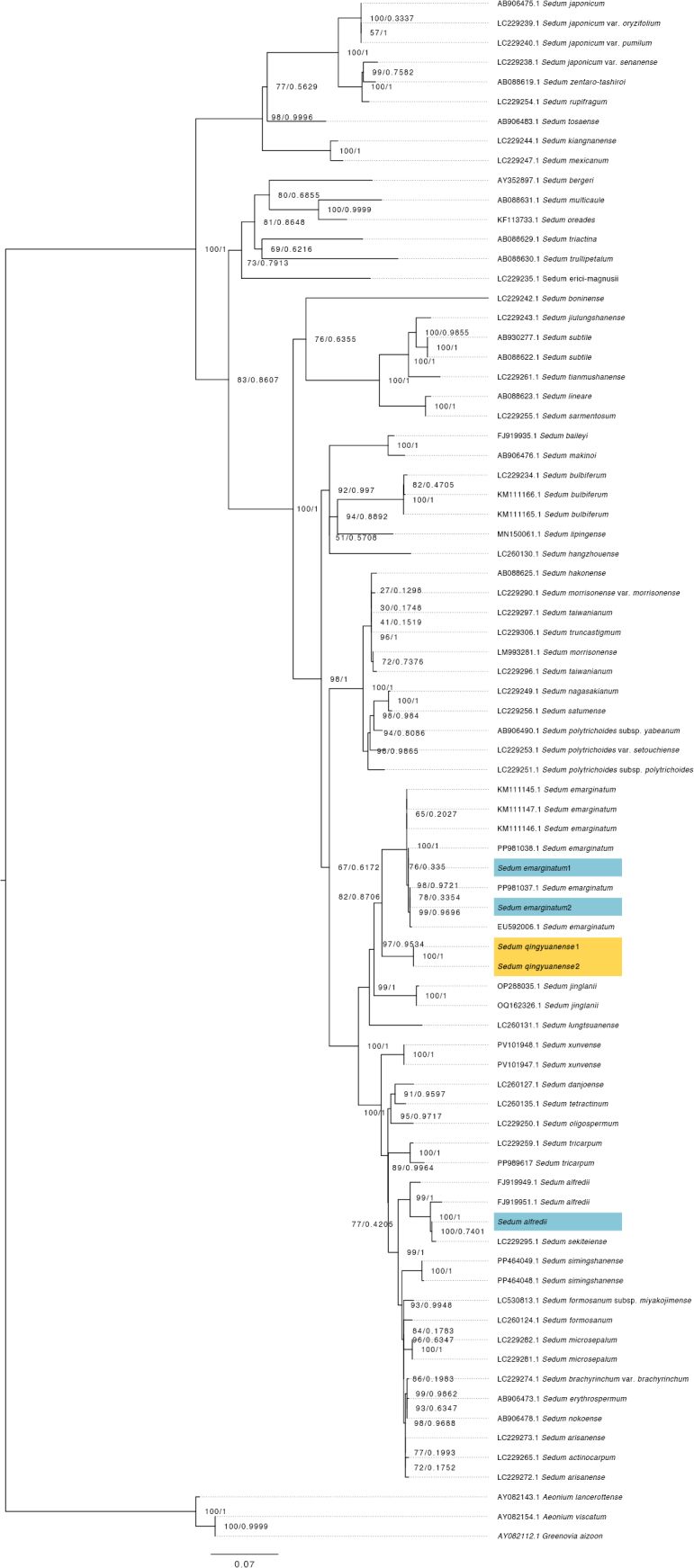
Maximum Likelihood phylogenetic tree constructed based on ITS sequences of *S. qingyuanense* and closely related species with Bayesian posterior probabilities mapped onto the branches. Node support values are given as bootstrap support value (BS) and Bayesian posterior probabilities (PP). *S. qingyuanense* is highlighted in yellow and bolded. The newly sequenced *S. alfredii* and *S. emarginatum* from this study are marked in blue.

The final length of the aligned ITS sequences was 659 bp. Using these ITS data, we successfully constructed a Maximum Likelihood (ML) tree (Fig. 1). In the ML tree, *Sedum qingyuanense* was clearly distinguishable from its morphologically similar relatives, *S. alfredii* and *S. emarginatum*. It formed a distinct clade, positioned as a sister group to *S. emarginatum*, while *S. alfredii* was located on a separate branch of the tree. Notably, although *S. qingyuanense* appeared phylogenetically close to *S. jinglanii* in the ML tree, it can be unequivocally distinguished from this species morphologically. Moreover, their habitats are markedly distinct: *S. jinglanii* is endemic to the Danxia landscape of Danxiashan Mountain in Shaoguan City, whereas *S. qingyuanense* is restricted to the granite cliffs in TaiHe Grotto of Qingyuan City.

This new species closely resembles *S. alfredii* morphologically and may have previously been misidentified as such. However, it can be differentiated by its smaller sepals, creeping sterile stems, opposite leaves, emarginate leaf apices, conspicuously pseudopetiolate, and consistently spatulate leaf shape. Because of its emarginate leaves and opposite phyllotaxy, *S. qingyuanense* may also be confused with *S. emarginatum*. However, the two species are readily distinguishable by differences in sepal size, leaf shape and size, presence of creeping sterile stems, and conspicuous pseudopetiolate. Morphological analyses of the putative new species and its relatives reveal that, despite some shared characteristics, they can be clearly distinguished by several diagnostic traits.

Based on our measurements and previous studies (Fu and Ohba 2001; Wu et al. 2012; Huang et al. 2023; Dai et al. 2025), we summarize and detail the morphological distinctions among these three species in Table 2.

**Table 2.** Morphological comparisons of *S. qingyuanense*, *S. alfredii*, *S. emarginatum*, *S. jinglanii*.

Character	Species			
	*S. qingyuanense*	*S. alfredii*	*S. emarginatum*	*S. jinglanii*
Fertile stems	4–15 cm	10–20 cm	10–27 cm	10–15 cm
Phyllotaxy	opposite, sometimes alternate	alternate	Opposite	opposite
Leaf apex	emarginate	obtuse sometimes emarginate	Emarginate	obtuse sometimes emarginate
Leaf blade	spatulate to broadly obovate	linear-cuneate, spatulate, or obovate	spatulate-obovate to broadly obovate	spatulate or obovate
Leaf size	1.1–5.9 × 0.5–1.4 cm	1.2–3 × 0.2–0.6 cm	1–2 × 0.5–1 cm	0.8–2.9 × 0.4–1.2 cm
Pseudopetiolate	conspicuously 8–24 mm	inconspicuously	Inconspicuously	inconspicuously
Sterile stem	creeping	ascending	absent	ascending
Sepal shape	linear-spatulate	linear-spatulate	lanceolate to narrowly oblong	linear-spatulate
Sepal size	1.8–3.2 × 0.5–1.5 mm	3–5 × 1–1.5 mm	2–5 × 0.7–2 mm	2–3.1 × 0.7–1.4 mm
Flowering	March–April	April–May	May–June	April–May

### ﻿Taxonomic treatment

#### ﻿ Sedum qingyuanense

Taxon classificationPlantaeSaxifragalesCrassulaceae

X.W.Yi, Z.Y.Xie & Q.Fan sp. nov.

CAD17E2F-C962-523D-AF55-E9EA300D0A35

urn:lsid:ipni.org:names:77373284-1

Figs 2, 3, 4, 5

##### Chinese name.

清远景天 (Qīng yuăn JĬng tiān).

##### Type.

China • Guangdong Province, Qingyuan City, TaiHe Grotto; on rocky cliff; 23.7553°N, 112.9807°E; 238 m a.s.l.; 18 April 2025; *X.W.Yi & K.Tang QY-1001* (holotype: SYS00237022).

**Figure 2.**
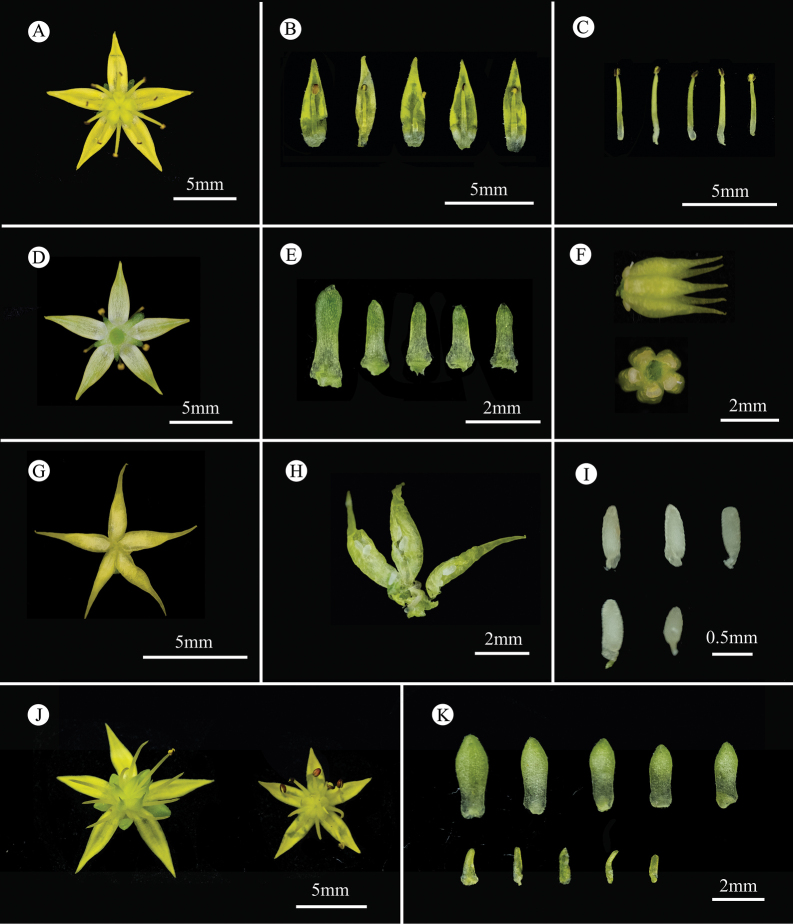
*Sedum qingyuanense*. **A.** Flower; **B.** Petals and epipetalous stamens; **C.** Episepalous stamens; **D.** Back view of flower (with sepals); **E.** Sepals; **F.** Frontal and lateral aspects of carpels; **G.** Unripe follicles; **H.** Opened follicles; **I.** Unripe seeds; **J.** Flower compared with *S. alfredii* (left: *S. alfredii*; right: *S. qingyuanense*); **K.** Sepals compared with *S. alfredii* (upper: *S. alfredii*; lower: *S. qingyuanense*). Photos: XiaoWei Yi.

##### Diagnosis.

The new species is distinguished from its congeners by the combination of creeping sterile stems, conspicuous pseudopetiolate, and small linear-spatulate sepals (1.8–3.2 × 0.5–1.5 mm). It differs from close species, *S. jinglanii*, *S. alfredii* and *S. emarginatum* in having creeping sterile stems (vs. ascending sterile stems), smaller sepals and more distinctly pseudopetiolate. In addition, we have also provided photographs of *S. emarginatum* and *S. alfredii* (Fig. 6).

**Figure 3.**
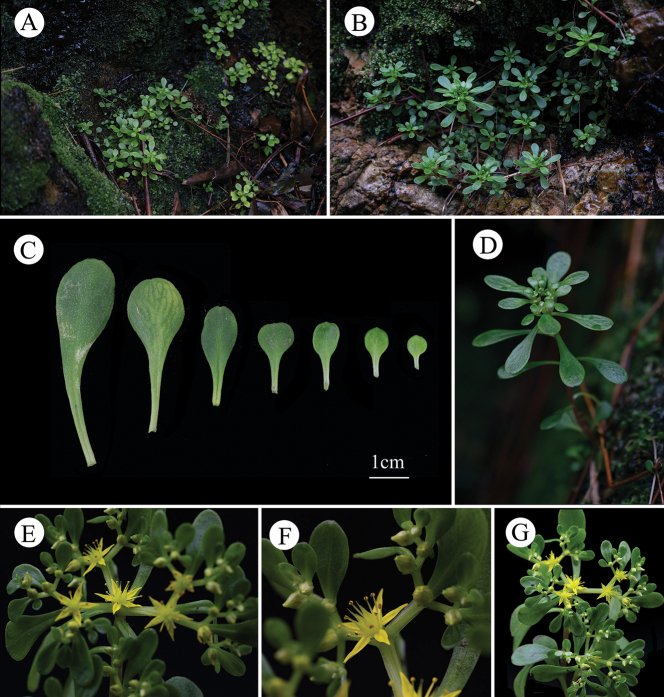
*Sedum qingyuanense*. **A–B.** Habitat; **C.** Leaves of a single *S. qingyuanense*; **D.** Immature cyme; **E–G.** Mature cyme. Photos: XiaoWei Yi & Ke Tang.

**Figure 4.**
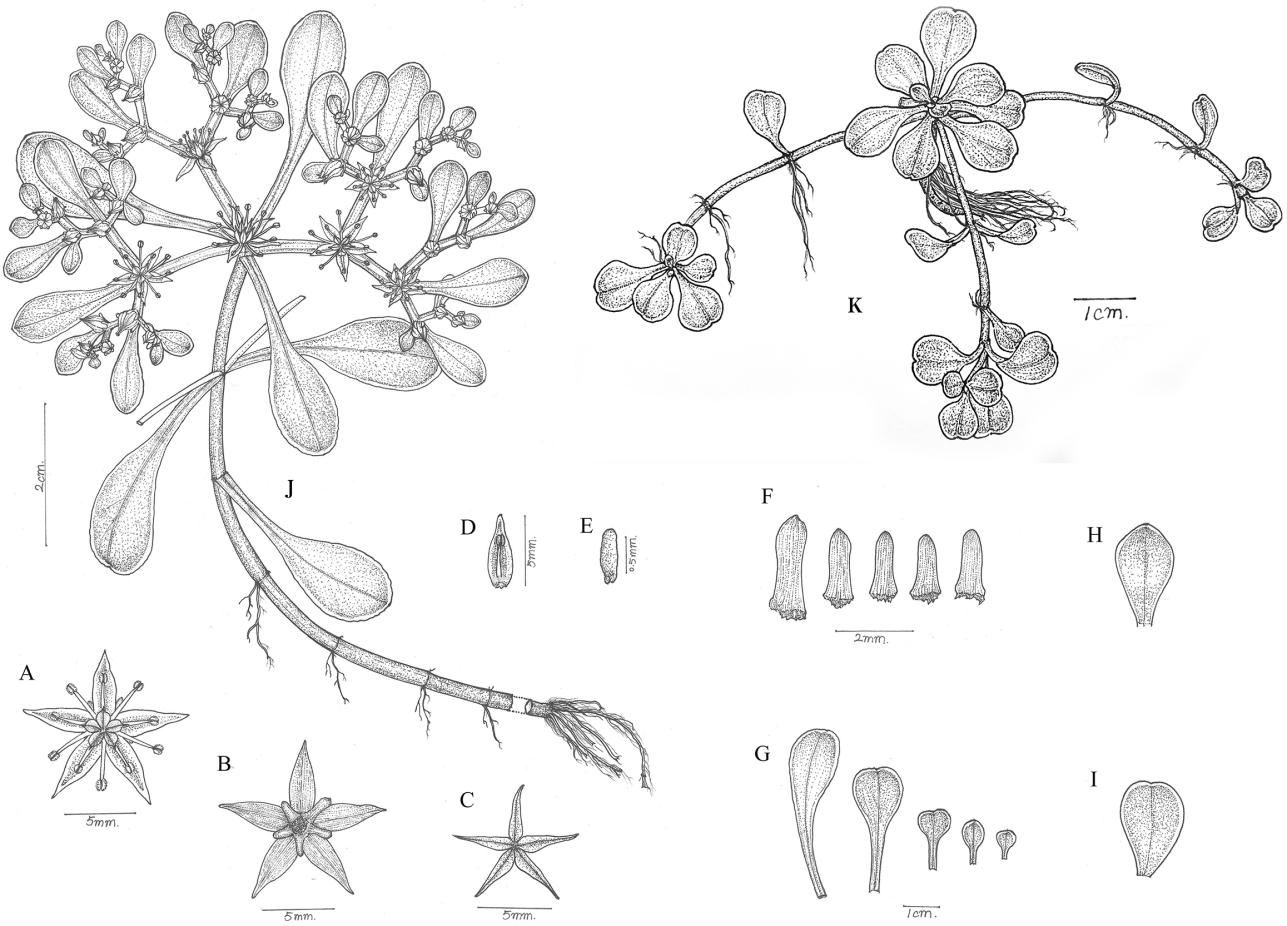
*Sedum qingyuanense*. **A–B.** Flowers; **C.** Follicles; **D.** Petal with stamen; **E.** Seed; **F.** Sepals; **G.** Leave; **H.** Bract; **I.** Basal leaf; **J.** Fertile stems; **K.** Sterile stems. Drawing by RongEn Wu.

##### Description.

Perennial herb, entirely glabrous. Stems succulent; fertile stems suberect 5–20 cm long, apex erect, usually 3-branched above; sterile stems present, creeping, rooting at nodes and producing new rosettes. Leaves opposite, conspicuously pseudopetiolate; leaf blades spatulate to broadly obovate, margin entire, apex emarginate, base attenuate with a spur, 1.1–5.9 × 0.5–1.4 cm. Cymes 3–8 cm in diameter, usually 3-branched, multiflowered; bracts obovate with an obtuse apex. Flowers sessile, 7–10 mm long, pentamerous, actinomorphic. Sepals 5, linear-spatulate, 1.8–3.2 × 0.5–1.5 mm, base spurred. Petals 5, yellow, lanceolate to lanceolate-oblong, 4–6 × 1.1–1.7 mm, apex acuminate, base connate ca. 0.3 mm. Stamens 10 antepetalous stamens ca. 3 mm long, adnate to petals for ca. 0.3 mm; antesepalous stamens ca. 4.5 mm long. Carpels 5, lanceolate, erect, connate at base, 3–4 mm long. Follicles obliquely divergent, many-seeded; placentation marginal. Seeds ovoid, brown at maturity, 0.6–0.8 mm long.

##### Phenology.

Flowering from March–April; fruiting in May.

##### Etymology.

The specific epithet refers to the distribution of this species in Qingyuan City.

##### Distribution and habitat.

The new species is endemic to Qingyuan City, Guangdong Province, southern China, growing on rocky cliffs at 200–300 m a.s.l.

##### Conservation status.

The Extent of Occurrence (EOO) and Area of Occupancy (AOO) were calculated following the guidelines of the International Union for Conservation of Nature (IUCN 2024). All known occurrences of *Sedum qingyuanense* are currently restricted to a single locality—TaiHe Grotto, Qingxin District, Qingyuan City, Guangdong Province, China. The EOO was estimated using the minimum convex polygon method based on this single site, resulting in an EOO < 2 km^2^. The AOO was assessed using a standard 2 km × 2 km grid system, and as all individuals fall within one grid cell, the AOO is inferred to be ≤ 4 km^2^. Given that the entire population occurs in a heavily visited tourist area along roadsides and is subject to ongoing anthropogenic threats (e.g., trampling, habitat disturbance), and considering its extremely limited distribution, *Sedum qingyuanense* meets the criteria for Critically Endangered (CR) under B1ab(iii)+2ab(iii).

**Figure 5.**
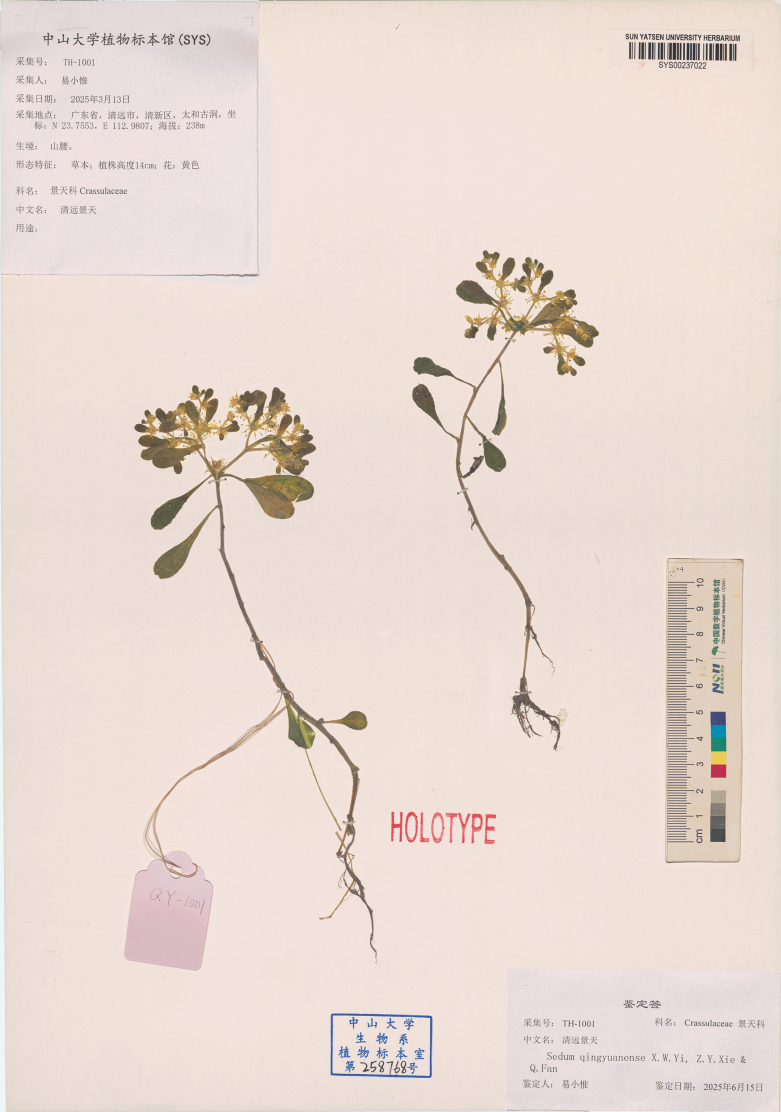
Holotype of *Sedum qingyuanense*.

**Figure 6.**
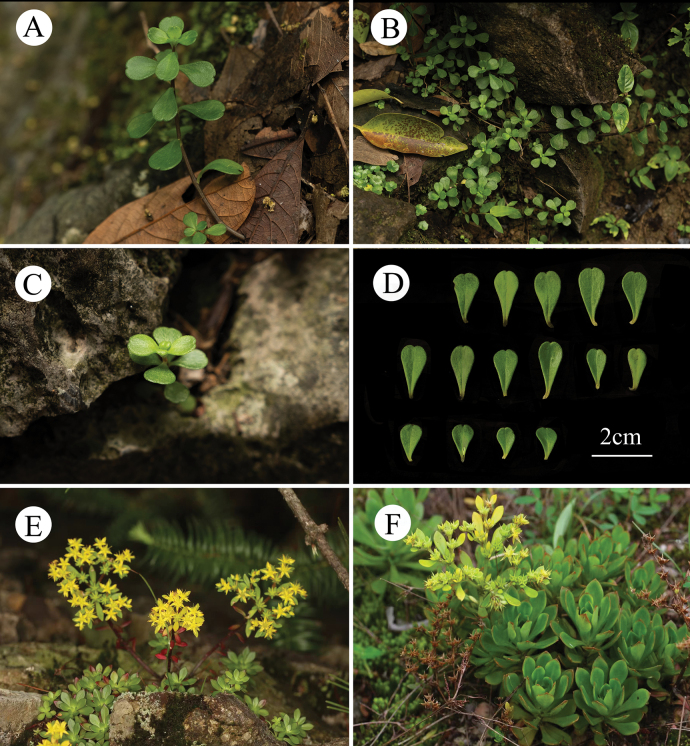
**A–D.***Sedum emarginatum* from its type locality (LingYin Temple, HangZhou, ZheJiang, China); **E, F.***S. alfredii*. Photos: YueLiang Xu.

## Supplementary Material

XML Treatment for Sedum qingyuanense

## ﻿Additional information

### Use of AI

No use of AI was reported.

### Author ORCIDs

Xiao-Wei Yi https://orcid.org/0009-0004-8867-7418

Zhi-Yi Xie https://orcid.org/0009-0004-4443-4502

Xue-Gui Shen https://orcid.org/0000-0002-5619-7784

Wei Xu https://orcid.org/0000-0003-2907-0478

Qiu-Gen Zeng https://orcid.org/0009-0003-2250-4648

Ke Tang https://orcid.org/0009-0009-9108-5145

Qiang Fan https://orcid.org/0000-0003-4254-6936

### Data availability

All of the data that support the findings of this study are available in the main text.

## Supplementary materials

Supplementary material 1 *Sedum qingyuanense* paratype Data type jpg https://binary.pensoft.net/file/1490216This dataset is made available under the Open Database License (http://opendatacommons.org/licenses/odbl/1.0/). The Open Database License (ODbL) is a license agreement intended to allow users to freely share, modify, and use this Dataset while maintaining this same freedom for others, provided that the original source and author(s) are credited.Xiao-Wei Yi, Zhi-Yi Xie, Xue-Gui Shen, Wei Xu, Qiu-Gen Zeng, Ke Tang, Yan-Shuang Huang, Qiang Fan
