# Supplementary figures and images for: ﻿Sedum qingyuanense (Crassulaceae), a new species from Qingyuan, Guangdong, China
Source: PhytoKeys. 2025 Dec 15;268:45–58. doi: 10.3897/phytokeys.268.174287 (PMC12723398; doi:10.3897/phytokeys.268.174287)

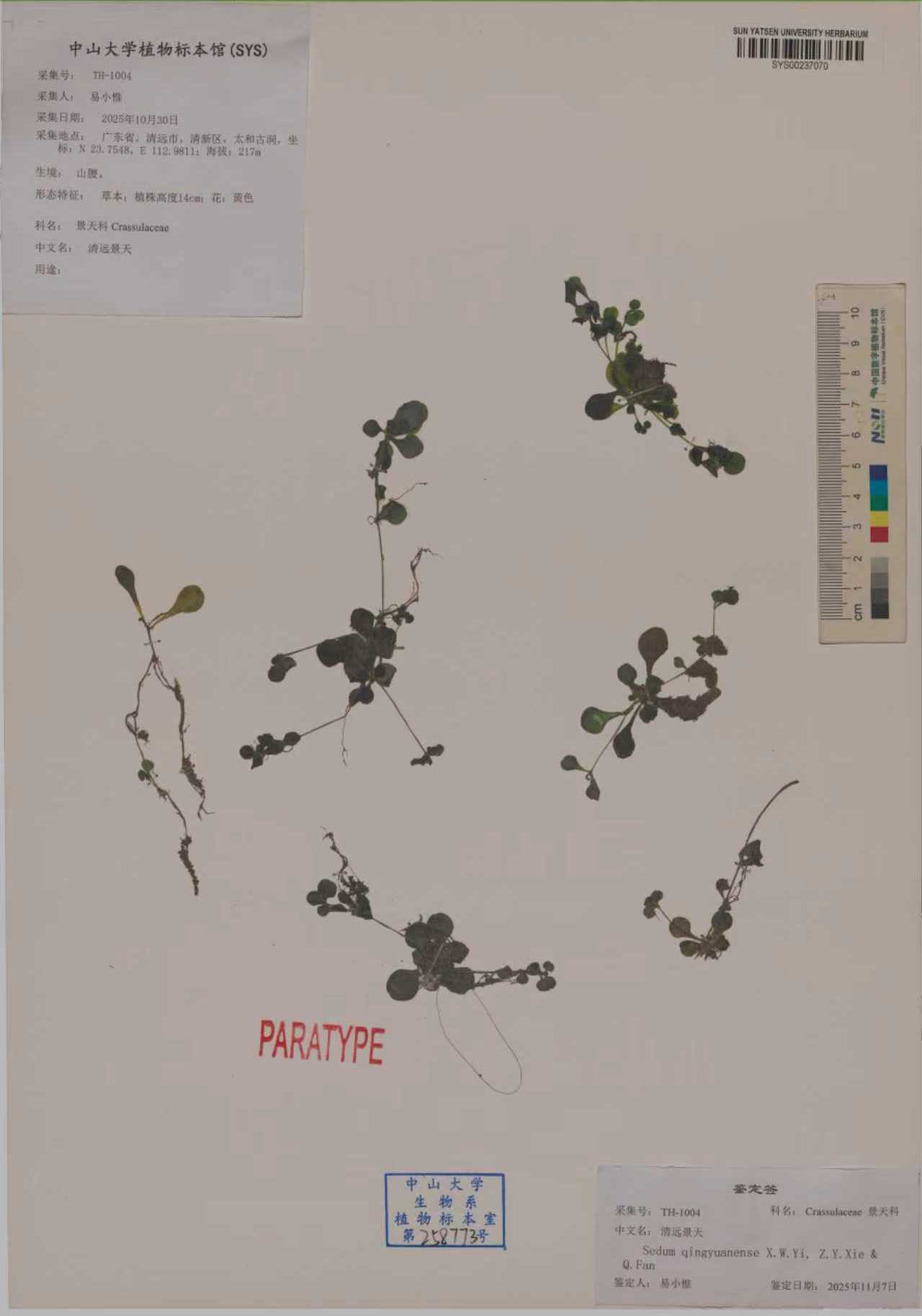

Supplement: Supplementary material 1 — Sedum qingyuanense paratype [file phytokeys-268-045_article-174287__-s001.jpg]
